# Tonotopic Organization in the Depth of Human Inferior Colliculus

**DOI:** 10.3389/fnhum.2013.00586

**Published:** 2013-09-19

**Authors:** David Ress, Bharath Chandrasekaran

**Affiliations:** ^1^Neuroscience Department, Center for Perceptual Systems, Imaging Research Center, Institute for Neuroscience, The University of Texas at Austin, Austin TX, USA; ^2^Department of Communication Sciences and Disorders, Center for Perceptual Systems, Institute for Neuroscience, The University of Texas at Austin, Austin, TX, USA

**Keywords:** audition, tonotopy, inferior colliculus, fMRI, midbrain

## Abstract

Experiments in animal models indicate that inferior colliculus (IC), the primary auditory midbrain structure, represents sound frequency in a particular spatial organization, a *tonotopy*, that proceeds from dorsal and superficial to ventral and deeper tissue. Experiments are presented that use high-resolution, sparse-sampling functional magnetic resonance imaging (fMRI) at 3 T to determine if tonotopic gradients can be reliably measured in human IC using high-resolution fMRI. Stimuli were sequences of bandpass-filtered noise with different center frequencies, presented sequentially while fMRI data were collected. Four subjects performed an adaptive frequency-discrimination task throughout the experiment. Results show statistically significant tonotopic gradients within both ICs of all subjects. Frequency gradients as a function of depth were measured using surface-based analysis methods that make virtual penetrations into the IC tissue. This organization was evident over substantial portions of the IC, at locations that are consistent with the expected location of the central nucleus of IC. The results confirm a laminar tonotopy in the human IC at 3 T, but with a heterogeneous, patchy character. The success of these surface-based analysis methods will enable more detailed non-invasive explorations of the functional architecture of other subcortical human auditory structures that have complex, laminar organization.

## Introduction

Organization by frequency, *tonotopy*, is one of the central organizing principles of the auditory system. The inferior colliculus (IC) is the primary auditory midbrain structure that forms a major convergence zone within the auditory system, receiving obligatory bottom-up connections from the brainstem auditory structures, and direct corticofugal feedback connections from auditory cortex. The IC consists of three prominent and functionally distinct subdivisions: the tonotopically organized central nucleus, the multisensory external cortex, and the dorsal cortex, which receives corticocollicular connections, primarily from the auditory cortex. Invasive studies on animal models show that the CN of IC exhibits tonotopic organization along the dorsal-to-ventral direction, which roughly corresponds to the depth direction within this laminated tissue (Schreiner and Langner, 1997; Malmierca et al., 2008; Baumann et al., 2011; Cheung et al., 2012). Electrophysiological studies in animal models show finely tuned topographical mapping of frequency along the laminar axis of the IC (Schreiner and Langner, 1997; Baumann et al., 2010). Similarly, anterograde labeling studies from the cochlear nucleus also show evidence for laminar frequency selectivity, but these results suggest a more heterogeneous, patchy distribution of frequency selectivity for the bottom-up inputs to IC (Oliver and Morest, 1984; Hofstetter and Ehret, 1992; Loftus et al., 2008).

In humans, the small size of the IC (<9 mm), coupled with the limited resolution of conventional human neuroimaging studies, have precluded the non-invasive examination of frequency representation. Further, subcortical structures are harder to image because of their susceptibility to physiological noise (Guimaraes et al., 1998). Conventional functional neuroimaging studies employ 3–10 mm sampling and spatial blurring, making it impossible to resolve functional subdivisions within the IC. In addition, the IC has a complex organization that represents multiple auditory features, with tonotopic specificity shown to be largely resolved along the laminar axis (Zook et al., 1985; Malmierca et al., 1993; Schreiner and Langner, 1997). The complicated internal structure of the IC requires a more detailed examination using high-resolution imaging and depth-resolved analyses.

Two recent studies have examined tonotopic organization using a resolution (2-mm FWHM) that is higher than traditional neuroimaging studies. Baumann et al. (2011) examined tonotopic and rate-dependent gradients in rhesus monkeys using a 4.7 T MRI scanner and found an orthogonal relationship between frequency and rate representation in the IC. More recently, De Martino et al. (2013) examined tonotopic organization in the human IC using a 7 T MRI scanner. Both studies showed a dorsolateral-to-ventromedial tonotopic gradient in the IC preferentially responding to low-to-high frequencies, respectively. While these studies demonstrate the utility of very high-field strengths in resolving tonotopic gradients within the IC, it is unclear whether clear tonotopy is evident at smaller field strengths. Functional neuroimaging studies at 3 T have demonstrated tonotopy in the human auditory cortex, in a manner consistent with the organization found in other primates (Barton et al., 2012; Moerel et al., 2012), suggesting the possibility that tonotopy can also be evidenced at 3 T.

**Figure 1:**
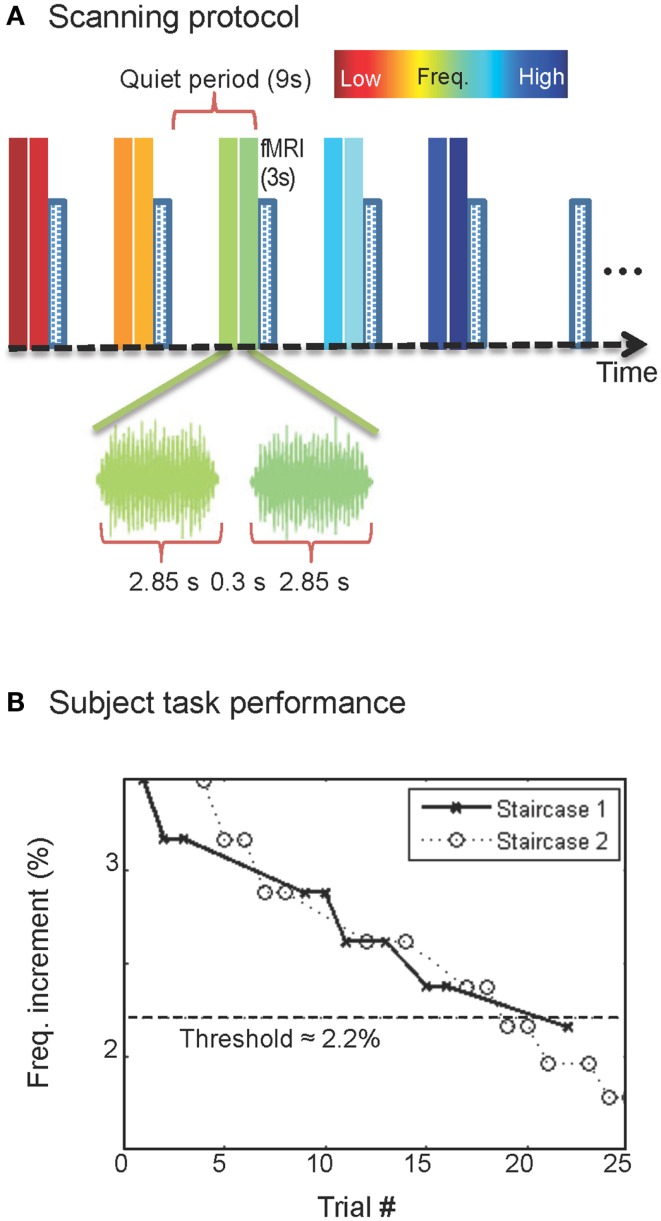
**Protocol, stimulus, and task**. **(A)** Sequence of stimulus presentation and fMRI acquisition; sound frequency is color-coded. **(B)** Typical subject performance during a single fMRI run.

The goal of this study is to examine frequency representation in the human IC using high-resolution (1.2 mm) functional magnetic resonance imaging (fMRI) at 3 T, adapted for auditory stimulation. During fMRI, subjects listened to sequences of five sounds in the range of 0.25–8 kHz, while performing a frequency-discrimination task (Figure 1). Surface-based analyses were then used to systematically evaluate tonotopy within human IC.

## Materials and Methods

### Subjects

Subjects were four male adults (age range 21–31), with normal hearing (air-conduction thresholds <20 dB nHL for octaves from 0.25 to 8 kHz). Informed consent was obtained from all subjects in accordance with procedures approved by the Institutional Review Board of The University of Texas at Austin.

### Stimulus and task

Auditory stimuli, presented through MRI compatible earphones, consisted of bandpass-filtered (0.6-octave bandwidth) white noise, presented with five different center frequencies, logarithmically spaced on 0.25–8 kHz (Figure 1A). The sounds were followed by a sixth quiet-period with no sound presented to act as a reference. This sequence was repeated five times during each 6-min run.

To ensure that participants paid consistent attention to the stimuli, they performed a challenging two-interval forced-choice frequency-discrimination task. Sounds were presented in pairs, each lasting 2.85 s with a 0.3-s gap. The sound in one of the two periods, randomly chosen, had a slightly higher center frequency than the other, and subjects used a MRI compatible button box after each sound pair to indicate their judgment as to which interval had the higher frequency. No response was given during the final quiet-period of each sequence. During stimulus periods, the frequency difference, typically 3–8%, between the two sounds was adjusted in real time using a pair of randomly interleaved two-up, one-down staircases. After each run, the subject’s current frequency-discrimination threshold value was estimated as the average frequency difference over the latter half of the trials in that run. Frequency difference for the next run was then initialized to a value 30% greater than this estimate. This creates a stable pattern of performance in which subjects perform very well on the task at the start of each run, and then steadily return to their threshold value (Figure 1B). In addition, subjects practiced the task in advance of scanning so that their performance was stable during the fMRI. Across runs, subjects’ performance was 75–85% correct.

### fMRI protocol

Stimulus presentations occurred every 12 s while high-resolution (1.2-mm cubic voxels) T2*-weighted images were collected in a sparse-sampling approach. In sparse-sampling designs, one attempts to sample the hyperoxic peak of the BOLD response evoked by preceding stimulus, then allocate a sufficient delay to allow that response to decay away before the next sample is obtained. Moreover, the delay is adjusted so that the nuisance response from the scanner-acquisition noise is minimized (Hall et al., 1999). The purpose of the long presentation period is to drive the desired stimulus-evoked response into a fairly stable “flat-top” so that it can be sampled by the fairly long 3-s-duration 3-shot acquisition that immediately follows each stimulation period. Our timing choice was guided by previous sparse-sampling approaches that have examined auditory processing in cortical and subcortical structures (e.g., Gaab et al., 2003; Wong et al., 2008; Chandrasekaran et al., 2012).

Imaging was performed on a 3 T scanner (GE Signa Excite HD) using an eight-channel head coil. Ten 1.2-mm-thick quasi-coronal slices were acquired sequentially over a 170-mm field-of-view during a 3-s period immediately following each sound pair. A set of T1-weighted structural images was obtained on the same prescription using a three-dimensional (3D) RF-spoiled GRASS (SPGR) sequence (15°flip angle, 0.78-mm pixels). These images produced good gray-white tissue contrast and were used to align the functional data to a segmented structural reference volume, described below.

Functional image acquisition used a 3-shot spiral trajectory (Glover and Lai, 1998) with TE = 40 ms and TR = 1 s that has provided good results in human midbrain (Katyal et al., 2010, 2012). Between acquisitions, T1 equilibrium was maintained by continual slice excitation, but acquisition and crusher gradients were suppressed to create quiet-periods for presentation of the audio stimuli. Noise was further reduced by setting slew limits for the excitation pulses to 30 T/m/s. The scheme resulted in a noise reduction of ∼15 dB during the quiet-periods. A similar approach was used previously with EPI acquisitions (Schwarzbauer et al., 2006).

The use of the three-shot spiral acquisition acts as a temporal anti-aliasing filter that substantially reduces high-frequency physiological nuisance effects, particularly cardiac pulsations. In single-shot acquisition, each fMRI slice is acquired very quickly (∼20–30 ms) compared to hemodynamic time scales, but the samples are widely spaced (e.g., 2–3 s). This results in aliasing of high-frequency fluctuations driven by cardiac pulse (>1 Hz) and respiration (∼0.25 Hz). In our multi-shot imaging, acquisition is now distributed over multiple brief sampling periods separated by the relatively short TR = 1 s. This preparation implements a low-pass filter upon the data. This avoided the use of cardiac gating, and also reduced respiratory artifacts (Ress et al., 2007; Katyal et al., 2010, 2012).

**Figure 2:**
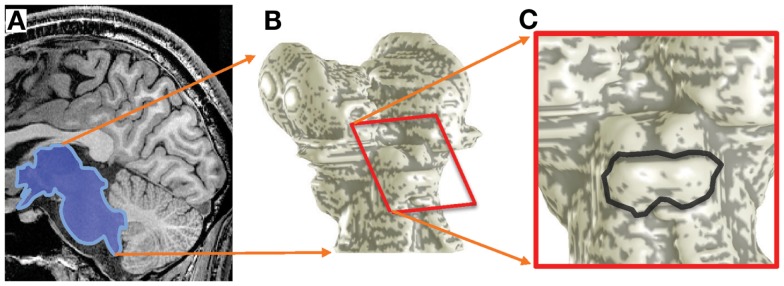
**Surface-model construction**. **(A)** MRI volumes obtained with 0.7-mm voxels were segmented to delineate the boundaries of brainstem tissue (blue). **(B)** A smooth surface was constructed at the segmentation boundary; gray overlay shows curvature. **(C)** Enlargement showing IC boundaries (black).

The same monotonically increasing sequence of five sound frequencies and quiet-period was repeated five times in each fMRI run (6-min duration, 120 fMRI volumes per run), and 10–12 runs were collected in each scanning session. The fMRI data were compensated for subject motion using a robust intensity-based scheme (Nestares and Heeger, 2000). Data were also corrected for spatial variations, introduced mostly by receiver-coil inhomogeneity, using a spatially smoothed homomorphic normalization to the temporal mean of the fMRI time series, and high-pass filtered to reduce the effects of slow image-intensity drifts. The anatomical images collected in each session were then used to align and transform the functional data to a structural 3D reference volume, which was acquired for each subject in a separate session (Figure 2A). The structural reference volume was T1-weighted with good gray-white contrast, acquired using a 3D, inversion-prepared, SPGR sequence (*T*_I_ = 450 ms, 0.7-mm isometric voxel size).

The noise in fMRI data is known to have a non-Gaussian distribution (Biswal et al., 1995; Holmes et al., 1997; Glover et al., 2000; Kruger and Glover, 2001). Typically, fMRI data is therefore “whitened” by performing a mixture of spatial and temporal blurring (Worsley and Friston, 1995; Friston et al., 2000). To maintain high spatial resolution, we wanted to avoid any blurring. Therefore, we used “bootstrapping,” a non-parametric method to estimate statistical significance. The bootstrapping procedure obtains a high-quality estimate of the statistical distribution of the observed data. The data, for example, the BOLD response from 55 presentations of a particular stimulus frequency, are resampled with repetition, and then averaged together. Each iteration of this procedure thus selects both a random subset of the original data, and a random set of weight factors. It can be shown analytically (Ephron and Tibshirani, 1998), that many repetitions of this procedure converges very nearly to the original distribution of the data. Thus, bootstrapping entirely avoids the need for assumptions of a particular, usually Gaussian, noise distribution. Instead, the actual distribution is estimated, and this distribution is used to directly calculate confidence intervals upon the data. For our data here, we resampled the data with replacement 1000 times; error bars then show the 68% confidence intervals on the bootstrapped distribution, which is similar to what would be presented for the standard-error-of-the-mean for a normal distribution.

### Image analysis

We segmented the brainstem tissue (Figure 2A) using the ITK-SNAP application (Yushkevich et al., 2006). A smooth surface was then interpolated at cerebrospinal fluid-tissue interface of the IC (Figure 2B) using a deformable-surface algorithm (Xu et al., 2006). This surface provided vertices and outward normal vectors used as a reference for the depth calculations described below as well as a means to visualize the functional data (Figure 2C). A nearest-neighbor Euclidean distance map was calculated between the IC tissue voxels and the vertices of the refined IC surface. Thus, each volume voxel was associated with a frequency response across the five tones, and a depth coordinate (Ress et al., 2007; Khan et al., 2011).

**Figure 3:**
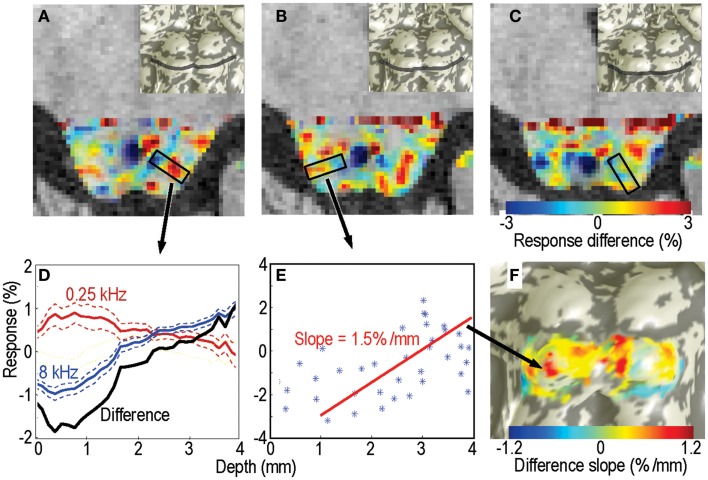
**Response differences between the highest and lowest frequency stimuli for subject 1**. **(A–C)** Response difference is shown as a color overlay upon axial high-resolution structural anatomy images of the IC. Transparency is modulated by statistical significance of the sound stimuli vs. quiet-periods. Insets show location of each slice on the IC surface model. **(D)** Responses as a function of depth to the lowest- (red) and highest-frequency stimuli (blue); difference is shown in black. These responses were obtained from a single depth penetration with a strong tonotopic gradient shown by the black rectangle in **(A)**. Thin dashed lines show 68% confidence intervals (e.g., SEM). **(E)** Response difference vs. depth for all voxels in another penetration marked in **(B)**. Red line is a linear fit the data beginning at 1-mm depth into the tissue. **(F)** The slopes of these linear fits are shown as a color overly upon this surface rendering of the subject’s IC.

As a first form of analysis, we calculated the BOLD response amplitude differences between the highest and lowest stimulus frequencies (Figure 3D). This procedure is analogous to that used by Baumann et al. (2011).

**Figure 4:**
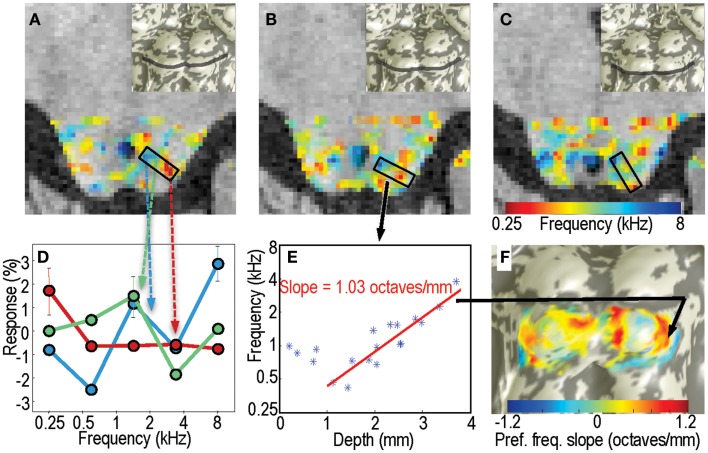
**Centroid frequencies in IC tissue**. **(A–C)** Color overlays of centroid frequency within IC, which vary from 0.25 kHz (red) to 8 kHz (blue). **(D)** Frequency response data for three sample voxels in a region in panel A. Line colors are color-coded in correspondence to the centroid frequency overlay. Red curve has a centroid near 0.3 kHz, green curve near 1.5 kHz, and blue near 3.5 kHz. **(E)** Centroid frequency for all voxels in the penetration marked by the black rectangle in **(B)**. A strong depth gradient that begins 1 mm below the surface is captured by the linear fit shown by the red line. **(F)** The linear fit slope data is marked as a color overlay upon the IC surface model.

As a second form of analysis, we characterized the frequency response for each voxel within the tissue of IC by calculating the centroid (first moment) of its frequency response (Figure 4D): $f_{\text{cent}}=\Sigma_{i}f_{i}A\left(f\right)∕\Sigma_{i}A\left(f_{i}\right)$, where *A*(*f_i_*) is the fMRI response amplitude to sound at frequency *f_i_*, and *i* indexes across the five frequencies. Amplitudes for each sequence of five stimuli were made positive definite by subtracting the minimum value. Because the difference between sound pairs was small (typically <5%), the frequency increment was neglected in these calculations. Each sound was repeated typically 55 times in a session. Using a bootstrapping approach as described above, we estimated confidence intervals on the centroid frequencies. To estimate tuning width we used the second moment of the amplitude response: $f_{\text{tune}}=\sqrt{\Sigma_{i}{\left(f_{i}-f_{\text{cent}}\right)}^{2}A\left(f_{i}\right)∕\Sigma_{i}A\left(f_{i}\right)}$.

We had originally planned to use a phase-encoded analysis approach, similar to what has been used in the measurement of visual retinotopy and tonotopy in cortex (Engel et al., 1997; Barton et al., 2012). However, the very slow period, 72-s, of each stimulus cycle made this analysis susceptible to low-frequency scanner drift noise. Although they were therefore noisier, the phase-encoding results were qualitatively similar to those obtained by the centroid-frequency approach described above.

We evaluated the gradients of both high-low response difference and centroid frequency with depth across the IC in a depth range of 0–5 mm. The notion that IC frequency preference is organized by depth is has been suggested by previous animal studies (e.g., Schreiner and Langner, 1997; Loftus et al., 2008), and the utility of such methods with high-resolution fMRI was demonstrated by our previous work (Katyal et al., 2010). The portion of the surface corresponding to IC, estimated based on positive tissue curvature, ranged from 110 to 182 mm^2^ across the four subjects. At every vertex in this portion (328–433 locations) on each IC surface model, we created normally oriented, 1.2-mm-diam cylinders. Specifically, we start from each surface vertex and create a patch corresponding to all surface vertices with manifold distances <0.6 mm from the starting vertex. These surface vertices are then extended along the inward-directed mean surface normal of the patch to penetrate the full depth of the tissue with a cylinder of constant diameter. We use these cylinders to probe the variation of response difference (Figure 3E) or centroid frequency (Figure 4E) with depth in the tissue, a hemodynamic analog to making electrode penetrations into the tissue. We then recorded the centroid frequency as a function of depth along the virtual penetration, and fit the result with a straight line; the slope of the fitted line, *g*, was an estimate of tonotopic gradient with depth. Linear fits were attempted after removing a variable amount of superficial tissue that varied from 0 to 1.5 mm, and the starting depth that yielded the best linear fit was chosen. Significance of the slope values was again obtained by a bootstrapping procedure. The amplitude-difference or preferred-frequency data obtained from each of 1000 resampled averages was fit as a function of depth by a straight line. The fraction of the resampled data sets with negative slopes provides an estimate of the *p* value for the gradient.

The orientations of those penetrations that corresponded to significant tonotopic depth gradients were used to obtain a measure of the mean tonotopic depth gradient. Specifically, we formed a weighted average: $\left\langle n\right\rangle =\Sigma_{i}g_{i}n_{i}∕\Sigma_{i}g_{i}$, where *n_i_* is the direction of each significant penetration *i*, having depth gradient *g_i_*. The resulting unit vectors yield two angles, one in the sagittal plane, and the other in the axial plane. We adopt the convention that an angle of 0°points directly anterior, with positive sagittal angles rotating superior, and positive axial angles rotating to the right.

## Results

The sparse-sampling scheme captured large amplitude changes that often exceeded 1% of the local mean. Response differences showed strong depth gradients in many portions of each IC that was studied (Figure 3). The gradients had a complex spatial distribution, but bands of low- and high-frequency responsive tissue could be seen on particular slices (Figures 3A–C). Virtual penetrations were made by constructing cylinders were normally oriented to the superficial surface of the tissue (shown schematically by black rectangles in Figures 3A,B). A thin band of overlaying tissue, consistent with the anatomic location of the external cortex, 0–1.5-mm thick, was observed in some regions, e.g., the lateral surface of the right colliculus in Figure 3B. Since tonotopy is not a feature of the external cortex of the IC, this region is ignored in the subsequent depth analysis.

Responses to the lowest and highest-frequency stimuli often showed opposing gradients with depth for many of the virtual penetrations, yielding a strong positive depth gradient in the high-low response difference (Figure 3D). The difference is initially noisy or constant as we penetrate through what we assume to be external cortex, then rises strongly. For each penetration, we fitted a straight line to the depth gradient after discarding the putative external cortex (Figure 3E). These virtual penetrations and linear fits were performed across the entire surface of both ICs in each subject. The slopes of the fits could then be visualized as color overlay upon the IC surface models (Figure 3F). Depth-gradient results for all subjects show regions of significant positive depth gradients across both ICs, with a patchy spatial organization very similar to that obtained from the frequency-centroid analysis presented below.

Figures 4A–C shows the spatial structure of centroid frequency on three axial slices in subject 1; examples from other slice orientations and subjects are shown in Figure S1 in Supplementary Material. Color overlay shows centroid frequency with the transparency modulated by the sound > quiet *p* value: voxels with *p* < 0.05 are opaque, logarithmically transitioning to transparent for voxels with *p* ≥ 0.5. Much like the amplitude-difference data, the preferred-frequency responses exhibit patchy bands of frequency selectivity.

Example voxel responses taken from a region with significant tonotopic depth gradients in centroid frequency illustrate their selectivity (Figure 4D). Superficial voxels often displayed low-frequency preference, with a distinct low-pass response characteristic. Similarly, deep voxels often had high-pass characteristics. Voxels at intermediate depths had mid-frequency preferences overall, but had more heterogeneous response patterns that often included a mixture of low- and high-frequency response.

The centroid frequency, *f*_cent_, was quite stable in all IC voxels. Bootstrapped confidence intervals had a mean value <0.5 octaves, and did not exceed 0.9 octaves. Thus, compared to the 5-octave frequency band of the stimuli, the experimental variability in centroid frequency was small, typically <±10%.

In portions of IC, the centroid frequency showed positive gradients with depth. Examples of virtual “penetrations” into the tissue clearly show the depth gradients (Figure 4E). The linear fit captured the depth gradient magnitude, which was then displayed upon the IC surfaces models (Figure 4F).

**Figure 5:**
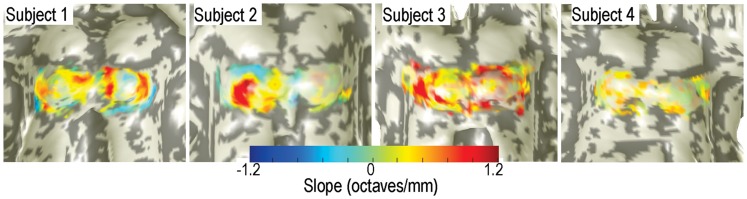
**Depth gradients of centroid frequency are displayed as a color overlay upon IC surface models of all four subjects**. Transparency is modulated by the statistical significance of the depth gradients.

Significant (*p* < 0.05) patches of tonotopic representation with depth were evident in all subjects (Figure 5). Significant gradients varied from 0.3 to 1.2 octaves/mm. The transparency of the depth-gradient color overlay is modulated by its statistical significance: the overlay is opaque for *p* < 0.05, and becomes more transparent as *p* approaches 0.5. The spatial distribution of the tonotopic gradients was complex and varied substantially amongst the four subjects. Gradients were typically stronger and more extensive on the left colliculus then on the right. Left-colliculus gradients were typically larger on the lateral and rostral surfaces, with less distinct gradients on the medial surface. Gradients were generally least significant over the very center of the colliculus. Weak but significant regions of reversed depth gradients were present around the edges of the ICs of subjects 1 and 2. The centroid-frequency depth gradients were somewhat stronger than those obtained by the high-low response differences described above, but both exhibited qualitatively similar spatial distribution across the IC surfaces.

Various features of the centroid-frequency data are summarized in Table 1. Total collicular area varied from 110 to 182 mm^2^. Areas of significant depth gradients varied from 19 to 32 mm^2^ (mean across subjects of 25 mm^2^), which was 18% of the total area of bilateral IC. About twice that area (53 mm^2^) showed trends (*p* < 0.16) toward depth gradients. Mean gradient was 0.53 octaves/mm, about half of what would be required to linearly resolve the full stimulus gamut in a 5-mm depth of tissue (see Discussion). Linear fits within each penetration were typically of high-quality, *R*^2^ ∼ 0.5. Mean tonotopic depth gradients for 3/4 subjects were inclined downward; subject 2 had a mean gradient pointing slightly upward. Three of the four subjects also displayed small rightward tilts to their mean gradients. The initial depth used to maximize the fit quality varied from 0.57 to 0.9 mm across subjects.

**Table 1:** **Summary of quantitative results from the tonotopic depth analysis**.

	Subject 1	Subject 2	Subject 3	Subject 4	Mean
Significant tonotopic area (*p* < 0.05, mm^2^)	30	19	32	19	25 ± 7
Trend tonotopic area (*p* < 0.16, mm^2^)	62	38	72	42	53 ± 16
Total area (mm^2^)	121	138	182	110	138 ± 31
Depth slope (octaves/mm)	0.59 ± 0.22	0.68 ± 0.19	0.49 ± 0.17	0.36 ± 0.13	0.53 ± 0.14
Sagittal plane angle	−22°	7°	−34°	−17°	−14°
Axial plane angle	11°	8°	21°	33°	−18°
Variance explained by linear fit	0.43 ± 0.20	0.62 ± 0.20	0.57 ± 0.21	0.37 ± 0.18	0.50 ± 0.12
Initial depth (mm)	0.57 ± 0.42	0.90 ± 0.46	0.72 ± 0.51	0.71 ± 0.55	0.73 ± 0.14

We examined the effects of spatial smoothing on our data by blurring the original fMRI data volumes with a 2-mm FWHM Gaussian kernel, then repeating the same depth analysis (Figure S2 in Supplementary Material). Significant positive depth gradients are still evident, with spatial structure largely similar to that observed in the original data. However, the blurring tends to reduce the absolute magnitude of the depth gradients, and increases the area of reversed gradients observed at the margins of the colliculi.

**Figure 6:**
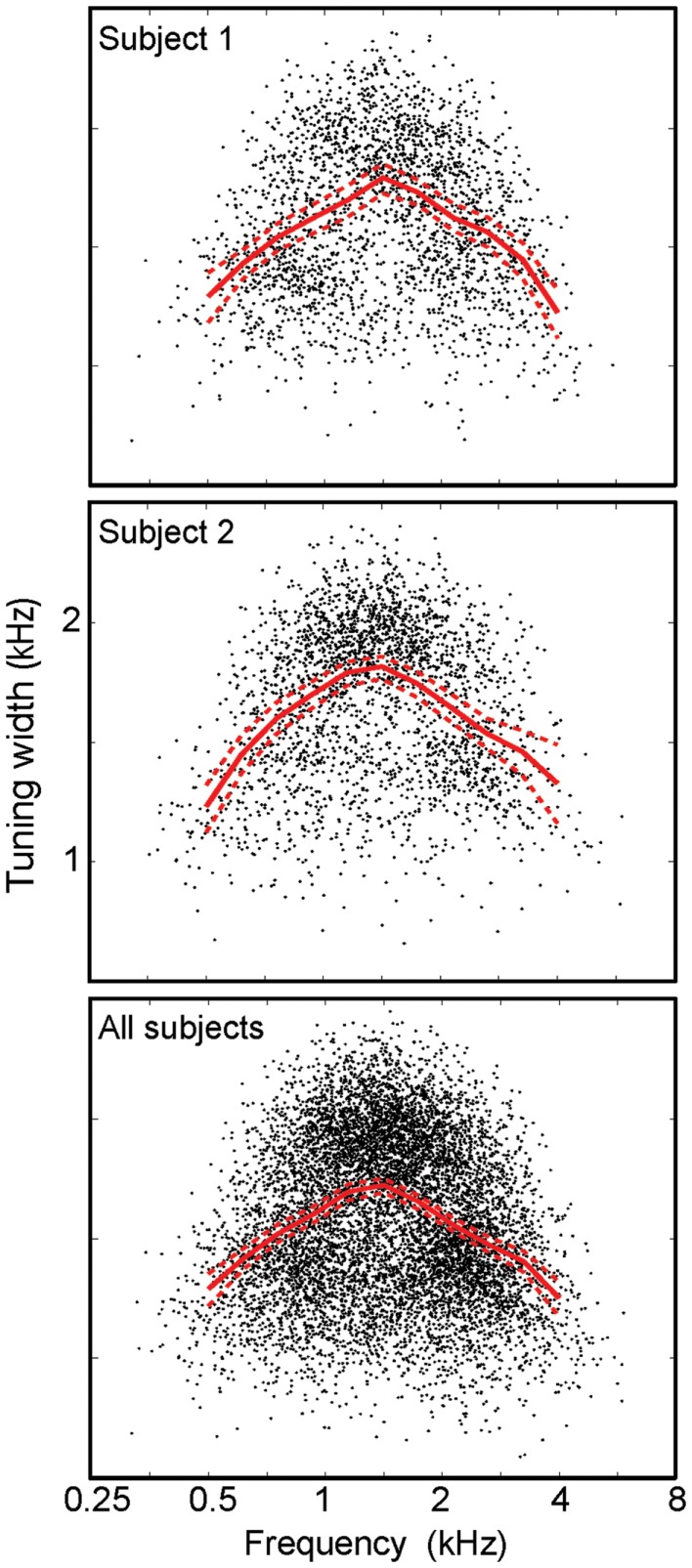
**Frequency tuning width vs. centroid frequency**. Points (black) for individual voxels plotted with a regridded average (red) with bootstrapped confidence intervals all display an “inverted-U” shape.

Frequency tuning was examined by plotting the tuning width as a function of centroid frequency (Figure 6). The results show an “inverted-U” shape for all individual subjects, with a similar profile for all subjects taken together. Each data point shows tuning width for a single IC voxel, while the red lines show a regridded average with bootstrapped 68% confidence intervals. On average, tuning widths are about 40% broader at intermediate frequencies than they are at the lower and higher ends of the stimulus frequency band. Bootstrapped reliability estimates indicate that tuning widths at both the lowest centroid frequencies (0.25–0.5 kHz) and highest centroid frequencies (4–8 kHz) are both significantly lower (*p* < 0.02) than tuning widths at intermediate frequencies (1.5–3 kHz) for each individual subject.

## Discussion

We used high-resolution imaging and surface-based analyses at traditional 3 T field strength to assess frequency representation within the human IC. Maps of centroid frequency showed a consistent depth-wise frequency organization in portions of both ICs of all subjects. To examine the laminar topography of the IC, we computationally modeled the surface of the IC, which permitted electrode-like “penetrations” into the depth of tissue. We observed significant tonotopy in the depth of IC tissue, with superficial layers showing greater preference to low frequencies and deeper layers to high frequencies. The depth gradients were evident by examining a simple subtraction of response to high-frequency stimuli from that evoked by low-frequency stimuli, or by evaluating centroid frequencies for each voxel sampled.

While several studies have documented midbrain activity to auditory stimulation (Guimaraes et al., 1998; Hawley et al., 2005; Sigalovsky and Melcher, 2006; Rinne et al., 2007; Chandrasekaran et al., 2012), these studies could not determine the complex 3D functional topography of the IC because of their limited spatial resolution. More recently, neuroimaging studies have used very high-field fMRI to evaluate tonotopy at the IC. Baumann et al. (2011) showed a dorsolateral-to-medioventral tonotopic gradient in rhesus monkeys at 4.7 T. Similar results were found in humans for 7 T imaging (De Martino et al., 2013). In the current study, the use of an auditory-optimized fMRI pulse sequence at a resolution of 1.2 mm enabled frequency analysis within the IC at 3 T field strength. In addition, surface-based analysis allowed the examination of functional variation in frequency preference across the laminar depth of IC, which can be viewed as a hemodynamic analog of electrode depth penetrations.

The mean depth gradient for 3/4 subjects was fairly well aligned to a vector pointing toward the neuraxis. Subject 2, with perhaps the weakest data of the four, displayed a mean gradient that was almost horizontal. All subjects also exhibited small left-right asymmetries in the gradient direction, probably reflecting a combination of lateral-medial and left-right asymmetries. Thus, the notion of using tissue depth as a reference frame was proven to work fairly well, despite the patchy character of the data. However, IC has a great deal more curvature than SC, so it is not surprising that we see some regions of reverse gradients at the margins of the colliculi, particularly on caudal regions where curvature is largest. Another drawback of the depth-penetration approach is that voxels in deeper regions tend to get more heavily resampled by adjacent penetrations because of curvature effects.

Our results obtained from humans using non-invasive high (1.2 mm) resolution are largely consistent with invasive animal measurements of frequency preference in CN (Schreiner and Langner, 1997) as well as high-field neuroimaging studies, which indicate that individual neuronal frequency preferences are organized systematically by depth. In contrast to these studies, the detailed spatial structure of our preferred-frequency measurements shows a patchy character. The patchy nature of the topographical representation in our study may reflect the different neural sensitivity of BOLD fMRI. Specifically, various experiments indicate that fMRI is more sensitive to neuronal inputs (Logothetis et al., 2001), or to local-circuit activity (Angenstein et al., 2009). Accordingly, our results may more closely reflect the patchy, somewhat interdigitated tonotopic organization of bottom-up neuronal inputs to IC from the cochlear nucleus, as reported by anterograde labeling studies (Oliver and Morest, 1984; Hofstetter and Ehret, 1992; Loftus et al., 2008).

The patchy character of our results disagree with the much more continuous spatial gradient of tonotopic selectivity observed by Baumann et al. (2011) and De Martino et al. (2013). The observed gradients, by construction, are oriented normal to the tissue surface, but the aggregate weighted mean shows an angle that is oriented more perpendicular to the neuraxis than the nearly superior-inferior gradient direction observed by De Martino et al. The observed structure is unlikely to be consequence of noise; they are a real feature of the data. Accordingly, they are likely either to reflect one or more neural or hemodynamic effects. On the neural side, the observed patchiness could be the consequence of task-related recruitment of sub-regions of IC tissue. Task demands such as selective attention have been shown to affect IC (Rinne et al., 2008), but such effects have not yet been evaluated at these fine spatial scales. The observed patchiness could also be hemodynamic in character, as related to the particular vascular compartment sensitivities of the BOLD response evoked at 3 T field strength as compared to higher fields (Gati et al., 1997; Yacoub et al., 2001; Duong et al., 2003; van der Zwaag et al., 2009). Specifically, at 3 T, only about half of the BOLD contrast corresponds to the extravascular component that is dominant in the capillary parenchyma, while half of the signal comes from the intravascular component in larger blood vessels. Although the relative contribution of venous and capillary signals was estimated to be similar at 3 and 7 T in cerebral cortex (van der Zwaag et al., 2009), the situation could be different in subcortical tissue. If so, the observed patchy structure may reflect stronger contrast from regions of IC where there are larger concentrations of draining veins, while little contrast is observed in other regions.

Our high-resolution data had comparatively high levels of thermal noise. Conventional fMRI studies operate where the thermal SNR is typically >100; our images had SNR ∼20–30. This noise could also have caused the observed patchiness of the results. To address the effects of thermal noise on our results, we performed a parallel analysis in which the data were first blurred with a 2-mm FWHM Gaussian spatial kernel. The post-blurring is expected to improve thermal SNR by the square root of the voxel-volume ratio, that is (2/1.2)^1.5^ = 2.2; this increase in SNR should greatly reduce the degradation of the thermal noise. The spatial structure of the results was qualitatively similar: a patchy distribution of tonotopically selective regions on the IC surface. This result indicates that the observed patchiness is a real feature of the data, and not the consequence of random noise. This similarity is also consistent with the bootstrapped evaluation of the data, which indicate the tonotopic gradients are significant only within the patchy regions presented in the results. The invariability of the tonotopy to the spatial resolution is also consistent with the results of higher-field fMRI studies (Baumann et al., 2011; De Martino et al., 2013).

In particular, we tend to observe weaker tonotopic depth gradients in the very center of the IC, an area expected to have a clearly organized depth tonotopy. There are at least three possible reasons for this lack of clear tonotopy. First, tonotopic inputs to this region may too finely interdigitated to be resolved by our measurement. If, in fact, the measured hemodynamic response were evoked more strongly by the inputs to this portion of the colliculus, then the heterogeneity of responses in each voxel would tend to blur the frequency selectivity. Second, it is again possible that local vascular characteristics modulate the fMRI responses, yielding weaker contrast in the central portions of each colliculus. Third, the task demands of our protocol may tend to selectively recruit IC circuits in the observed sub-regions. Further experiments will be necessary to investigate these issues. In particular, it will be important to investigate the repeatability of the observed patterns across sessions.

Our stimulus protocol utilized a repetitive, monotonic cycle of stimulus frequencies. This choice was motivated by our original interest in a phase-encoded analysis (Engel et al., 1997; Barton et al., 2012), but has several drawbacks when used in conjunction with the current approach. In particular, the undershoot of the HRF evoked by each stimulus will impact the hyperoxic peak of the subsequent stimuli. However, the monotonic sequence somewhat softens this effect: the 0.25-kHz response undershoot will reduce the response to the 0.59-kHz stimulus, the 0.59-kHz response undershoot will reduce the response to the 1.41-kHz stimulus, and so forth. Thus, the undershoot acts as a form of blurring in the frequency domain, with relatively benevolent effects.

We did not observe narrow frequency tuning in our voxels, particularly at intermediate frequencies. The most likely explanation for this result is the spatial resolution of our measurements. Each voxel encompasses a volume of ∼1.7 μL containing several million neurons, so some heterogeneity of response can be expected. Interestingly, we observed sharper tuning at low and high frequencies, suggesting that the population response for these frequency bands is more spatially homogeneous than at intermediate frequencies. Also, the observed depth gradients did not span the full range of the stimuli. Again, this is likely the consequence of the available spatial resolution. Sharply tuned responses from both the highest and lowest frequency stimuli cannot be resolved, so the preferred-frequency depth gradients show a smaller range. Nonetheless, we do resolve the presence of a tonotopic gradient as a function of depth, and bands of tissue with clearly evident preferences for low, intermediate, and high frequencies.

Tonotopic depth gradients were particularly evident in the left colliculus of all subjects, but were weaker in the right colliculus for three of our four subjects. There are two possible reasons for these left-right asymmetries. First, they may be a consequence of purely vascular effects. In our superior colliculus neuroimaging work (Katyal et al., 2010), we found that visual responses for a particular subject were frequently much greater in one colliculus or the other, without any obvious neural correlate. Second, such asymmetries (relatively stronger leftward bias) could be a consequence of the task used in this study. Auditory tasks that require attentive processing are known to show a left-hemispheric bias (Bryden et al., 1983) and can modulate IC activity via corticocollicular connections (Rinne et al., 2007, 2008). Corticocollicular connections are largely ipsilateral (from the auditory cortex) (Winer, 2006), and connect A1 neurons to neurons in the IC that have a similar frequency profile (Lim and Anderson, 2007). Thus, it is possible that the left-dominant collicular activity could reflect corticocollicular effects. Future studies need to compare the effects of task (e.g., compare passive listening to active task), and to evaluate the role of top-down corticocollicular pathways in determining frequency selectivity within the IC.

Examining the IC encoding of frequency information is of significant clinical relevance. The IC has been considered as an alternate site for implant in hearing-impaired individuals who have contraindications for cochlear implants. The IC implant was developed based on animal models that suggest tonotopic organization in the CN. In fact, a few patients have already received IC implants and their success has been varied (Lim and Anderson, 2007). A recent study showed that initial frequency resolution in an individual with an IC implant was poor, but more precise selectivity was found 4 months after implantation (Lim et al., 2013). While our data demonstrates tonotopic representation as a function of depth, we find that tonotopic gradients are patchy and variable across individuals, which could pose a significant challenge for the placement of midbrain implants. Accordingly, further studies using high-resolution fMRI could provide a critical tool for surgical planning.

Our high-resolution surface-based fMRI methods and results provide a foundation for further non-invasive experimental work in small auditory subcortical structures. The ability to localize frequency selectivity with the depth of IC opens up new frontiers in auditory neuroscience, permitting the quantification of functional response in humans using complex stimuli and experimental paradigms. Moreover, the high-resolution sparse-sampling also enables investigations in other brainstem nuclei such as the cochlear nucleus and superior olivary complex. For clinical uses, these results open up the use of readily available 3 T MRI scanners for surgical planning and other treatment options. In conclusion, our methods are a significant advance for high-resolution fMRI research of auditory function in general, and will permit a host of critical auditory neuroscience experiments in small subcortical auditory structures that have been inaccessible to conventional auditory fMRI studies.

## Conflict of Interest Statement

The authors declare that the research was conducted in the absence of any commercial or financial relationships that could be construed as a potential conflict of interest.

## Supplementary Material

The Supplementary Material for this article can be found online at: http://www.frontiersin.org/Human_Neuroscience/10.3389/fnhum.2013.00586/abstract

Figure S1 **Additional color overlays of centroid frequency on anatomy slices**. **(A,B)** Sagittal and coronal views of subject 1, respectively. **(C–E)** Sagittal, coronal, and axial views of subject 3. Inset surface figures show slice locations on surface models of each subject’s midbrain.

Figure S2 **Depth gradients obtained from data blurred with 2-mm FWHM kernel**. Same format at Figure 5.
