# Supplementary material for: Tonotopic Organization in the Depth of Human Inferior Colliculus
Source: Front Hum Neurosci. 2013 Sep 19;7:586. doi: 10.3389/fnhum.2013.00586 (PMC3776955; doi:10.3389/fnhum.2013.00586)
Supplement: Figure S1 — Additional color overlays of centroid frequency on anatomy slices. (A,B) Sagittal and coronal views of subject 1, respectively. (C–E) Sagittal, coronal, and axial views of subject 3. Inset surface figures show slice locations on surface models of each subject’s midbrain. [file 54808_Ress_DataSheet1.PDF]

Supplementary Figures

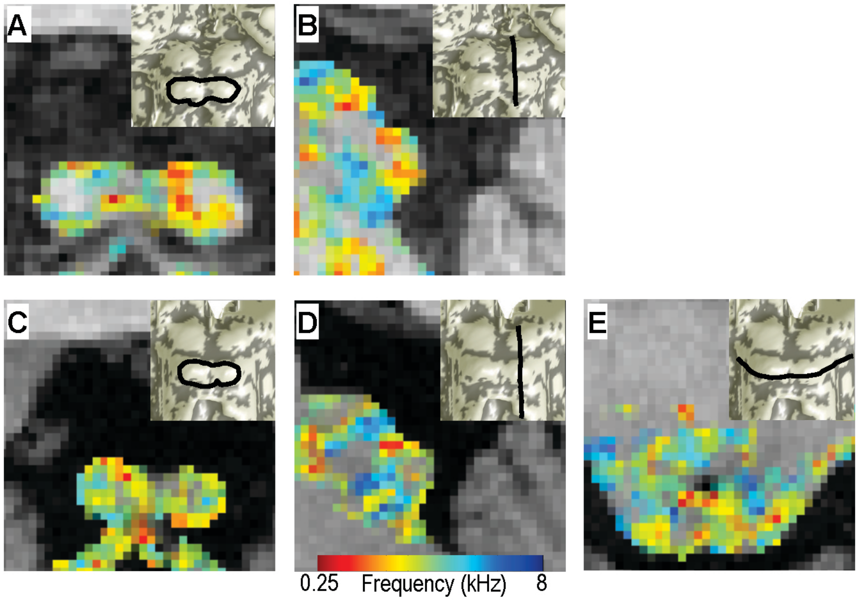

Supp. Figure 1: additional color overlays of centroid frequency on anatomy slices. (A, B) Sagittal and coronal views of subject 1, respectively. (C, D, E) Sagittal, coronal, and axial views of subject 3. Inset surface figures show slice locations on surface models of each subject's midbrain.

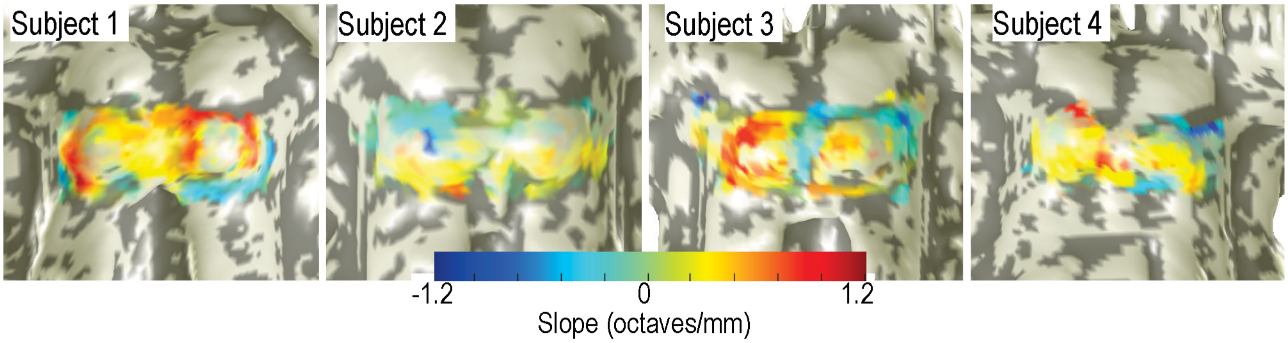

Supplementary Figure 2: depth gradients obtained from data blurred with 2-mm FWHM kernel. Same format at Fig. 5.
